# Prevalence and burden of gastrointestinal parasites in cattle and buffaloes in Jabalpur, India

**DOI:** 10.14202/vetworld.2016.1214-1217

**Published:** 2016-11-09

**Authors:** Priyanka Marskole, Yamini Verma, Alok Kumar Dixit, Madhu Swamy

**Affiliations:** 1 Department of Veterinary Pathology, College of Veterinary Science & Animal Husbandry, Nanaji Deshmukh Veterinary Science University, Jabalpur - 482 001, Madhya Pradesh, India; 2 Department of Veterinary Parasitology, College of Veterinary Science & Animal Husbandry, Nanaji Deshmukh Veterinary Science University, Jabalpur - 482 001, Madhya Pradesh, India

**Keywords:** buffalo, cattle, eggs/oocysts per gram, gastrointestinal parasites, prevalence

## Abstract

**Aim:**

The study was conducted to determine the prevalence and burden of gastrointestinal (GI) parasites in cattle and buffaloes of Jabalpur, Madhya Pradesh.

**Materials and Methods:**

The presence of helminths eggs and coccidial oocysts in fecal samples were detected using standard qualitative and quantitative methods. Identification of eggs or oocysts was done on the basis of morphology and size of the eggs or oocysts.

**Results:**

Out of 120 cattle and buffaloes examined, 73.33% were found positive for eggs of one or more species of GI parasite. The prevalence of parasitic infection was higher in cattle (75%) as compared to that of buffaloes (70.45%), but the difference was nonsignificant (p>0.05). Sex wise prevalence of GI parasites was higher in males as compared to that of females, but the difference was nonsignificant (p>0.05). The animals above 2 years of age were more affected by GI parasites as compared to animals of 6 months - 2 years of age, but the age wise differences were nonsignificant (p>0.05). Single parasitic infections were more common than mixed infections. The monthly prevalence of GI parasites in cattle and buffaloes were highest in the month of September (81.81%) and least in December (61.11%). The eggs/oocysts per gram in most of the animals, was in the range of 201-300.

**Conclusion:**

GI parasites are problem in cattle and buffaloes of Jabalpur, Madhya Pradesh. The prevalence rate of GI parasites varied with month. The burden of parasitic infection was moderate in most animals warranting treatment.

## Introduction

Parasitic infections are one of the major constraints for profitable dairy industry in tropical and subtropical countries including India. Gastrointestinal (GI) parasites cause considerable global economic losses as a consequence of reduced weight gain, digestive disturbance, lowered production, impaired reproductive performance, condemnation of affected organs, and mortality in infected animals [1]. In addition, the diverse agroclimatic conditions, animal husbandry practice, and pasture management largely determine the incidence and severity of various parasitic diseases in certain area.

In Madhya Pradesh, studies have been undertaken to provide information on the prevalence of GI infections in cattle and buffaloes [2,3], but limited attempt has been made to study prevalence and burden of GI parasitic infections in cattle and buffaloes in Jabalpur region [4,5]. Therefore, it is important to recognize, control, and prevent parasitic infection with better management and knowledge on the prevalence of GI parasitic infections.

This paper describes the prevalence and burden of GI parasites in cattle and buffaloes of Jabalpur District and its adjoining areas.

## Materials and Methods

### Ethical approval

The research was conducted after approval of Institutional Ethical Committee.

### Study area

The study was conducted in Jabalpur district of Madhya Pradesh. It is situated in between latitude 23° 10’ N and longitude 79° 56’ E with an average height of 411 m above mean sea level. Jabalpur has a humid subtropical climate typical of North-Central India (Madhya Pradesh and Southern Uttar Pradesh). During the study period, Jabalpur received an annual rainfall of 1143.30 mm. The maximum temperature varied from 21.2°C to 40.2°C and minimum 7.7°C to 26.8°C. The maximum relative humidity varied from 46% to 89% and minimum 18-67%.

### Study animals

The study was conducted on 120 animals (76 cattle and 44 buffaloes) of small holder dairy farms of Jabalpur. Animals were categorized according to sex, i.e., male and females and age, i.e., animals of 6 months to 2 years and >2 years. The zero grazing management system was followed in which animals were permanently housed, and fodder was obtained by cutting from various places and carried back to the housed animals. Concentrates and mineral supplementation were also given to growing and milking animals as per requirements. Anthelmintic treatments are on individual farm basis based on availability of money and drugs.

### Collection and examination of fecal samples

Fresh fecal samples of 120 animals (76 cattle and 44 buffaloes) were collected randomly from different localities of Jabalpur during August 2014 to March 2015. The fecal samples were collected directly from the rectum of individual cattle and buffalo using sterilized hand gloves and were processed. Floatation technique was used for demonstrating nematode and cestode egg, as well as oocyst of coccidia and sedimentation technique, was used for detecting the trematode eggs. The ova/eggs of parasites were identified from their morphological characters. Eggs/oocysts per gram (EPG/OPG) of infection were determined by modified McMaster technique [6].

### Data analysis

The data gathered from the study for the prevalence of parasitic infection were analyzed by Chi-square test as described by Snedecor and Cochran [7].

## Results and Discussion

Out of 120 cattle and buffaloes examined, 88 (73.33%) were found positive for eggs of one or more species of GI parasite. The prevalence of parasitic infection was higher in cattle (75%) as compared to that of buffaloes (70.45%), but the difference was nonsignificant (p>0.05). The prevalence of GI infections found in this study is much higher than the findings of Kashyap *et al*. [8] who reported 40.30% prevalence of GI helminths in cattle and buffaloes of Malwa region of Madhya Pradesh. Whereas, this finding is more or less similar to the earlier finding of Gupta *et al*. [5] who recorded 68.93% overall prevalence in cattle and buffaloes, 73% in buffaloes and 65% in cattle of same locality, whereas Mir *et al*. [9] reported 51.21% overall prevalence of GI parasitism in cattle and buffaloes, 38.70% in buffaloes and 67.15% in cattle of Jammu region. The variation in the findings with earlier reports might be due to the difference in the number of fecal samples examined, a period of study and geoclimatic conditions (temperature and humidity, etc.) that favors the survival of infective stage of the parasites and of intermediate hosts, managemental conditions and deworming practices. However, higher prevalence in cattle compared to buffaloes may be attributed to differences in feeding habit and habitats of the two species.

Overall, sex wise prevalence of GI parasites (Table-1) was higher in males (83.33%) as compared to that of females (70.83%), but the difference was nonsignificant (p>0.05). In cattle also, the prevalence of GI parasites in males (80%) was higher than females (74.24%) but the difference was nonsignificant (p>0.05). Similarly, in buffaloes, the prevalence was nonsignificantly higher (p>0.05) in males (85.71%) as compared to females (63.33%). The higher percentages of infection in male cannot be explained exactly but it might be due to the neglected attitude of the farmers toward the management of male animals. These findings are in agreement with Fikru *et al*. [10], Bilal *et al*. [11], and Awraris *et al*. [12] from different corners of the world.

**Table 1:** Sex wise and age wise prevalence of GI parasites in cattle and buffaloes.

Species	Factor level							

	Sex				Age			
	
	Male		Female		6 months-2 years		>2 years	

	Examined	Positive (%)	Examined	Positive (%)	Examined	Positive (%)	Examined	Positive (%)
Cattle	10	8 (80)	66	49 (74.24)	24	16 (66.67)	52	41 (78.85)
Buffalo	14	12 (85.71)	30	19 (63.34)	16	10 (62.50)	28	21 (75)
Overall	24	20 (83.34)	96	68 (70.83)	40	26 (65)	80	62 (77.50)

GI=Gastrointestinal

Further, the data were analyzed on the basis of age group (Table-1) and it was noted that the animals above 2 years of age were more affected (77.50% cattle and buffaloes, 75% buffaloes, and 78.84% cattle) by GI parasites as compared to animals of 6 months to 2 years of age (65% cattle and buffaloes, 62.50% buffaloes and 66.66% cattle), but the age wise differences were nonsignificant (p>0.05). The increase in the prevalence of GI parasites with the age has also been reported by Quershi and Tanveer [13] and Telila *et al*. [14]. On the contrary, Regassa *et al*. [15] stated that the younger animals are more susceptible than adult animals. The causes of variation in the prevalence of parasites in different age groups are difficult to explain but it might be due to an immune status of animals, difference in the grazing area and management conditions.

In this study, single parasitic infection was observed in 45.84% cattle and buffaloes, 47.73% buffaloes, and 44.75% cattle, while mixed infection was observed in 27.50% cattle and buffaloes, 22.73% buffaloes, and 30.26% cattle. The results for the parasite wise prevalence of GI parasites are presented in Table-2. Among nematodes, the prevalence rates were strongyle 60%, 47.73%, and 39% in cattle and buffaloes, buffaloes, and cattle, respectively, *Strongyloides* spp. (2.50%, 2.28%, and 2.64%), *Toxocara vitulorum* (1.67%, 0%, and 2.64%), and *Trichuris* spp. (1.67%, 2.28%, and 1.32%). The finding of predominant strongyle eggs in large ruminants was in agreement with Biu *et al*. [16] and Swarnakar *et al*. [17]. In trematodes, the prevalence of *Fasciola gigantica* was 4.17%, 0%, and 6.58% and amphistome was 5%, 4.55%, and 5.27% in cattle and buffaloes, buffaloes, and cattle, respectively. Higher prevalence of *Fasciola* spp. (4.44%) and amphistome (11.06%) in cattle and buffaloes was reported by Swarnakar *et al*. [17] while Gupta *et al*. [5] found 6.77% *F. gigantica* and 45% amphistome in buffaloes, and 0.99% *F. gigantica* and 17.59% amphistome in cattle. The only cestode observed in this study was *Moniezia* spp. with the prevalence of 7.5% in cattle and buffaloes, 6.82% in buffaloes and 7.90% in cattle. The prevalence of cestode was in line with the findings of Swarnakar *et al*. [17] and Keyyu *et al*. [18]. However, a higher prevalence was reported by Hailu *et al*. [19] and lower prevalence by Gupta *et al*. [5]. The variation in the prevalence might be due to the opportunity of exposure to the intermediate host and the free-living soil mites on pasture. *Eimeria* spp. was 28.34%, 29.55%, and 27.64% in cattle and buffaloes, buffaloes, and cattle, respectively. The prevalence rate was more or less similar with the findings of Haque *et al*. [20] and Gupta *et al*. [5].

**Table 2:** Parasite wise prevalence of GI parasites in cattle and buffaloes.

Parasite/species (%)	Cattle (n=76)	Buffalo (n=44)	Overall (n=120)
Strongyles	39 (51.32)	21 (47.73)	60 (50)
*Strongyloides* spp.	02 (2.64)	01 (2.28)	03 (2.50)
*T. vitulorum*	02 (2.64)	0	02 (1.67)
*Trichuris* spp.	01 (1.32)	01 (2.28)	02 (1.67)
*F. gigantica*	05 (6.58)	0	05 (4.17)
Amphistome	04 (5.27)	02 (4.55)	06(5)
*Moniezia* spp.	06 (7.90)	03 (6.82)	09 (7.5)
*Eimeria* spp.	21 (27.64)	13 (29.55)	34 (28.34)

*T. vitulorum=Toxocara vitulorum, F. gigantica=Fasciola gigantica*, GI=Gastrointestinal

The monthly prevalence of GI parasites (Table-3) in cattle and buffaloes were highest in the month of September (81.81%) and least in December (61.11%). According to Hailu *et al*. [19] the overall prevalence of GI helminths infection was high in October (wet month) and low in February. The reason for this could be conduciveness of the environmental condition for the development of larvae. The EPG/OPG of feces was considered to be the estimation of the burden of infection of GI parasites in cattle and buffaloes. The EPG/OPG in most of the fecal samples was in the range of 201-300 (Table-4). The EPG in the most of the studied animals indicated moderate infection that warrants treatment.

**Table 3:** Month wise prevalence of GI parasites in cattle and buffaloes.

Months	Cattle and buffaloes	Positive (%)
August 2014	10	07 (70.00)
September 2014	11	09 (81.81)
October 2014	14	11 (78.57)
November 2014	20	15 (75.00)
December 2014	18	11 (61.11)
January 2015	18	13 (72.22)
February 2015	14	10 (71.42)
March 2015	15	12 (80.00)
Total	120	88 (73.33)

**Table 4:** EPG/OPG of GI parasites in cattle and buffaloes.

Parasites	100-200			201-300			301-600		
		
	Overall	Buffalo	Cattle	Overall	Buffalo	Cattle	Overall	Buffalo	Cattle
Strongyle	14	04	10	33	13	20	13	04	09
*Strongyloides* spp.	01	01	00	02	00	02	00	00	00
*T. vitulorum*	01	00	01	01	00	01	00	00	00
*Trichuris* spp.	00	00	00	02	01	01	00	00	00
*Eimeria* spp.	07	03	04	12	04	08	15	06	09
*Moniezia* spp.	01	01	00	07	02	05	01	00	01

*T. vitulorum=Toxocara vitulorum*, EPG/OPG=Eggs/oocysts per gram

## Conclusion

Cattle and buffaloes in Jabalpur were infected with helminths and coccidia. The prevalence rates of GI parasites varied with month. The EPG of infection was moderate in most animals warranting treatment. Future studies are required to evaluate the economic impact of GI parasites in the study area.

## Authors’ Contributions

PM went to the field to collect the samples and carried out the research. YV and MS supervised the overall research work. AKD wrote the first draft before being revised and approved by all the authors.

## References

[1] Raza A.M, Iqbal Z, Jabbar A, Yaseen M (2007). Point prevalence of gastrointestinal helminthiasis in ruminants in southern Punjab, Pakistan. J. Helminthol.

[2] Kumar S, Das G, Nath S (2013). Incidence of gastrointestinal parasitism in buffalo in Central Madhya Pradesh. Vet. Pract.

[3] Jamra N, Das G, Haque M, Singh P (2014). Prevalence and intensity of strongyles in buffaloes at Nimar region of M.P. Int. J. Agric. Sci. Vet. Med.

[4] Agrawal M.C, Vohra S, Gupta S, Singh K.P (2004). Prevalence of helminthic infection in domestic animals in Madhya Pradesh. J. Vet. Parasitol.

[5] Gupta A, Dixit A.K, Dixit P, Mahajan C (2012). Prevalence of gastrointestinal parasites in cattle and buffaloes in and around Jabalpur, Madhya Pradesh. J. Vet. Parasitol.

[6] Zajac A.M, Conboy G.A (2012). Veterinary Clinical Parasitology.

[7] Snedecor G.W, Cochran W.G (1994). Statistical Methods.

[8] Kashyap Z, Sisodia R.S, Shukla P.C (1997). Incidence of gastrointestinal parasites in cattle and buffaloes in Malwa region of Madhya Pradesh. Haryana Vet.

[9] Mir M.R, Chishti M.Z, Rashid M, Dar S.A, Katoch R, Kuchay J.A, Dar J.A (2013). Point prevalence of gastrointestinal helmintheasis in large ruminants of Jammu India. Int. J. Sci. Res.Publ.

[10] Fikru R, Teshale S, Reta D, Yosef K (2006). Epidemiology of gastro intestinal parasites of ruminants in Western Oromia Ethiopia. Int. J. Appl. Res. Vet. Med.

[11] Bilal M.Q, Hameed A, Ahmad T (2009). Prevalence of gastrointestinal parasites in buffalo and cow calves in rural areas of Toba Tek Singh, Pakistan. J. Anim. Plant Sci.

[12] Awraris T, Bogale B, Chanie M (2012). Occurrence of gastro intestinal nematodes of cattle in and around Gondar town, Amhara regional state, Ethiopia. Acta Parasitol. Glob.

[13] Qureshi A.W, Tanveer A (2009). Seroprevalence of fasciolosis in buffaloes and humans in some areas of Punjab, Pakistan. Pak. J. Sci.

[14] Telila C, Abera B, Lemma D, Eticha E (2014). Prevalence of gastrointestinal parasitism of cattle in East Showa Zone, Oromia regional state, Central Ethiopia. J. Vet. Med. Anim. Health.

[15] Regassa F, Sori T, Dhuguma R, Kiros Y (2006). Epidemiology of gastrointestinal parasites of ruminants in Western Oromia, Ethiopia. Int. J. Appl. Res. Vet. Med.

[16] Biu A, Maimunatu A, Salamatu A.F, Agbadu E.T (2009). A fecal survey of gastrointestinal parasites of ruminants on the university of Maiduguri research farm. Int. J. Biomed. Health Sci.

[17] Swarnakar G, Bhardawaj B, Sanger B, Roat K (2015). Prevalence of gastrointestinal parasites in cow and buffalo of Udaipur district, India. Int. J. Curr. Microbiol. Appl. Sci.

[18] Keyyu J.D, Kassuku A.A, Msalilwa L.P, Monrad J, Kyvsgaard N.C (2006). Cross-sectional prevalence of helminth infections in cattle on traditional, small-scale and large-scale dairy farms in Iringa district, Tanzania. Trop. Anim. Health Prod.

[19] Hailu D, Cherenet A, Moti Y, Tadele T (2011). Gastrointestinal helminth infections in small-scale dairy cattle farms of Jimma town, Ethiopia. Ethiop. J. Appl. Sci. Technol.

[20] Haque M, Singh J, Singh N.K, Juyal P.D, Singh H, Singh R, Rath S.S (2011). Incidence of gastrointestinal parasites in dairy animals of Western plains of Punjab. J. Vet. Parasitol.

